# Shifting the centre of gravity: Towards a truly global representation in autism research

**DOI:** 10.1177/13623613231214644

**Published:** 2023-11-20

**Authors:** Gauri Divan, Chung-Hsin Chiang, Michele Villalobos, Muideen Bakare, Rosa A Hoekstra

*This editorial and the accompanying Special Issue is dedicated to the memory of our valued colleague Dr Muideen Owolabi Bakare, who unexpectedly passed away just prior to the publishing of this editorial.*

The vast majority of autism research papers published in the international peer-reviewed literature are conducted in high-income countries (HIC), particularly in North America and Europe (Franz et al., 2017). This is despite the fact that the majority of autistic children and those with other neurodevelopmental disabilities live in low-and middle-income countries (LMIC; Global Research on Developmental Disabilities Collaborators, 2018), often referred to as the Global South. This special issue titled ‘Autism Research in a Global Context’ aims to address this disparity by creating a platform that brings together high-quality studies from countries traditionally poorly represented in published literature.

As a team of five guest editors, three of us from the Global South, we were pleasantly surprised by the overwhelming response to our original request for abstracts, which reflected the thirst for such targeted calls and the barriers that may exist to researchers in the Global South. Over 80 abstracts were submitted, of which we finally accepted 19 manuscripts for publication. Given our different geographies and time zones, we were able to leverage the increasing ease of online connectivity for our editorial work, collaborating closely to reach editorial decisions. Our distinct time zones also meant challenging boundaries (including many calls outside normal working hours) and that sometimes not all of us could be present during meetings. It made us set up a system of concise and transparent communications allowing collaborative decision making. This process has been a tremendous exercise and learning experience for each of us on how editorial work should also reflect collaborative partnerships that we would like to see reflected in research. Through this editorial, we would like to share our reflections on our experience.

First, we have realised and experienced that the publication pathway for a researcher in the Global South is still mostly controlled by actors from the Global North: even if they work in LMIC settings, most journal editors are from HIC. This also applies to *Autism*, a journal that has only recently appointed its first editor from the Global South. Moreover, the majority of reviewers on journals’ records are also HIC based. For this special issue, we tried to invite experienced reviewers from a range of regions and countries, including researchers in Japan, Taiwan, India, Malaysia, Argentina, Chile, Brazil, Nigeria, Ethiopia, South Africa and China. We felt this was important to ensure that reviewers understood the contexts and complexities of working in LMIC settings when reviewing manuscripts for this special issue. This step means this special issue acts as a lever to increase the diversity of the reviewer pool for the journal going forward. We urge journals that truly want to represent the underserved populations of the world to act to expand representation at all stages within their editorial pipelines. We acknowledge this is not an easy task. For example, LMIC-based clinicians who are also researchers tend not to have protected research time and therefore often struggle to find time for editorial duties.

Second, as editors of this special issue, we set formal guidelines around author representation in submitted manuscripts. We wanted to ensure our special issue would not include examples of parachute research (The Lancet Global Health, 2018), where HIC researchers drop into a country, collect data and return home to write a paper without involving or acknowledging the contribution of local researchers or experts. To our delight, our special issue guideline to include representation of authors from the county where the work was conducted is now an explicit requirement of *Autism*, articulated in the journal’s revised *aims and scope* (https://journals.sagepub.com/aims-scope/aut). We consider this a significant success of this editorial partnership.

Third, while we stipulated that at least one author for each accepted publication should be from the context where the work was done; we noted that many of the articles submitted and those accepted for publication had additional author representation affiliated with HIC institutions, particularly in senior or corresponding author roles. We hope that these will increasingly become roles of mentorship, capacity building and guidance, with LMIC authors gradually taking more prominent roles in developing the ideas of research relevant for their contexts, gaining independent research funding from international funders and in drafting and submitting manuscripts independently. Health research and policy formulations in many LMIC are still largely focused on communicable and infectious diseases, rather than neurodevelopmental disabilities and autism (Olusanya et al., 2023). To increase the capacity for autism research worldwide and nurture a global research base at various stages of maturation, we advocate a model of inter-continental and intra-continental mentorship where more senior autism researchers from either context mentor those in earlier stages of their career and create space for the growing cadre of global researchers to become research leaders themselves. This also means creating space for LMIC researchers to be first authors rather than have middle authorship (Kelaher et al., 2016) and acknowledging the value of knowledge produced by local experts (Bhakuni & Abimbola, 2021). Capacity building for these roles can be supported by mentoring initiatives like the INSAR Early Career Mentoring programme (https://www.autism-insar.org/page/ECCMentorIng), though the issue of protected research time will continue to remain a challenge unless funders build in mechanisms to support these activities. An example is the National Institute for Health and Care Research’s Global Health Research funding mechanism (https://www.nihr.ac.uk/explore-nihr/funding-programmes/global-health.htm), mandating equitable partnerships between HIC and LMIC collaborating institutions and stipulating requirement for the HIC institute to support doctoral candidates in partner LMIC.

Fourth, the range of manuscripts submitted to us and finally reaching publication reflect the range of interests in autism research in LMIC, and the various stages that autism research may be in across different settings. This included research in exploring autism community priorities for autism research and capacity building in low-resource contexts (Lee et al., 2022; Perez Liz et al., 2023), developing and evaluating interventions (e.g. Brandi Gomes Godoy et al., 2023; Schlebusch et al., 2022) and applying novel technologies to facilitate autism assessment (Lockwood Estrin et al., 2023; Mukherjee et al., 2022). It is also interesting to see how research is being replicated across countries and regions, recognising that contextual factors play an important role (Garcia Torres et al., 2023; Zerihun et al., 2023). We are particularly proud to include in this special issue papers that report on research conducted in some of the world’s most challenging circumstances (Taresh et al., 2023).

Fifth, with English being the international language of research publication, researchers from the Global South are at a disadvantage. Most LMIC researchers do not speak English as their native language, and language editing services are expensive and out of reach for most. For this special issue, the journal publisher offered us editors the option to refer a manuscript for language editing free of charge. This allowed us to evaluate manuscripts purely based on the scientific content and not on adherence to standard English. The publisher is now more widely piloting this approach in its suite of journals to consider if this policy can be sustainably implemented longer term.

Finally, to attain research equity, it is important that research findings are accessible to all. There are two critical barriers to this: first, that scientific articles remain behind paywalls for most researchers in the Global South, isolating them from the latest research in their field; and second, fees for open-access publishing are prohibitively high for most LMIC researchers. Without open-access options, the copyright of the paper reporting on science generated in the LMIC setting is owned by the high-income setting journal. For this special issue, the *Autism* publisher agreed to make the articles freely available by waiving the usual open-access publication fee, so the work can be read globally. We call on journal publishers and editors to consider this issue of equity more fully. There are examples of journals that waive publication fees for LMIC-based corresponding authors; this is a model more publishers should consider.

We were delighted to edit this special issue and showcase the richness of autism research conducted across the globe. The interest in our special issue and the breadth of papers that made the final selection demonstrates that the centre of gravity of autism research is slowly shifting: global autism research is here and intends to stay. We look forward to seeing this momentum grow further over the next decade. We are confident that with implementation of some of the suggestions offered in this editorial, our field can move to the next step, from research conducted globally to research *led* by researchers globally.



**Gauri Divan**
Sangath, India

**Chung-Hsin Chiang**
National Chengchi University, Taiwan

**Michele Villalobos**
The University of Utah, USA

**Muideen Bakare**
Childhood Neuropsychiatric Disorders Initiatives, Nigeria

**Rosa A Hoekstra**
King’s College London, UK

